# Oncolytic Vaccinia Virus Expressing HSP70 shRNA Exerts Anti-Tumor Effects in Human Ovarian Cancer via Triggering the Autophagy–ROS Feedback Loop and Immune Activation

**DOI:** 10.3390/v17111423

**Published:** 2025-10-27

**Authors:** Zheqi Cai, Zhiyun Hong, Guohui Zhang, Tinwei Zhu, Yanrong Zhou, Ting Ye, Gongchu Li, Kan Chen

**Affiliations:** College of Life Sciences and Medicine, Zhejiang Sci-Tech University, Hangzhou 310018, China; 13017777880@163.com (Z.C.); 2023210901021@mails.zstu.edu.cn (Z.H.); h15382360260@163.com (G.Z.); z199720@outlook.com (T.Z.); zhouyanrong@zstu.edu.cn (Y.Z.); yeting@zstu.edu.cn (T.Y.)

**Keywords:** oncoVV-shHSP70, ovarian cancer, ROS-autophagy feedback, immune activation, humanized mouse model

## Abstract

Heat shock protein 70 (HSP70) represents a promising target for cancer therapy. Oncolytic vaccinia virus (oncoVV) mediates tumor regression through direct oncolysis and immune activation. However, the anti-tumor potential of HSP70-silenced oncoVV (oncoVV-shHSP70) remains unexplored. Here, we demonstrate that oncoVV-shHSP70 achieves superior tumor regression in ovarian cancer models (cell lines, immunodeficient mice and humanized mice) via dual mechanisms including enhancing apoptosis, autophagy flux, ROS generation, and immune reprogramming. Notably, we found that oncoVV-shHSP70 triggers an autophagy–ROS feedback loop that amplifies viral replication and pro-inflammatory cytokine expression. Crucially, in humanized mice, oncoVV-shHSP70 induced spatial redistribution of cytotoxic T cells, expanding tumor-infiltrating hCD8^+^hGZMB^+^ populations. These findings position oncoVV-shHSP70 as a promising viro-immunotherapy that co-opts HSP70 silencing to potentiate both direct oncolysis and anti-tumor immunity, providing a preclinical rationale for viro-immunotherapy in solid tumors.

## 1. Introduction

Ovarian cancer remains the most deadly gynecologic cancer, with over 70% of diagnoses occurring at advanced stages where typical treatments often falter due to immune evasion and chemoresistance [1,2,3]. Oncolytic virus (OV) therapy has surfaced as a promising approach because of its dual capability to destroy tumor cells directly and activate antitumor immunity [4,5,6,7]. Oncolytic adenovirus research was jump-started by ONYX-015, whose E1B-55 kDa deletion made it the first of its class to enter human studies [8,9]. The concept later yielded H101, an E1B/E3-deleted strain approved in China as the world’s first oncolytic virus therapy for nasopharyngeal carcinoma via intratumoral injection [10]. Building on these advances, H103, a type-2 recombinant that overexpresses heat-shock protein 70, was developed to boost both direct tumor lysis and HSP70-driven immune responses against primary and metastatic disease [11]. Among various oncolytic viruses, the vaccinia virus, belonging to the poxvirus family, has undergone extensive clinical evaluation. Its large double-stranded DNA genome (~190 kb) can accommodate significant transgenes (up to 25 kb), which facilitates the creation of armed, tumor-selective vectors [12]. The replication occurring in the cytoplasm helps mitigate concerns regarding insertional mutagenesis [13]. Prominent clinical candidates include Pexa-Vec (JX-594) aimed at patients with hepatocellular carcinoma [14] and vvDD-GFP, which has been tested in models of breast, colon, and ovarian cancers [12].

Our research team has previously developed a range of oncolytic vaccinia viruses (oncoVV) with enhanced antitumor effectiveness [15,16,17]. For instance, we created oncoVV-shSTRIP1, a recombinant vaccinia virus lacking thymidine kinase (TK) and silencing STRIP1, which received NMPA approval in 2023 for Phase I trials (GC001, CXSL2200569) targeting late-stage solid tumors. The classical view holds that oncolytic viruses eradicate tumors through high-level replication and direct cytotoxicity [18,19]. Emerging evidence, however, underscores the equal importance of indirect mechanisms: viral infection elicits endoplasmic-reticulum stress, releasing PAMPs that ignite innate immunity and elevate cytokines such as IFN-α, IFN-γ, and TNF-α. We previously demonstrated that the recombinant vaccinia virus oncoVV-AVL (engineered to express Aphrocallistes vastus lectin) lyses human ovarian cancer cells while augmenting autophagy and raising intracellular ROS [20]. These results underscore the diverse mechanisms by which oncolytic viruses exert anti-cancer actions [18,21].

Heat shock proteins (HSPs) are vital molecular chaperones crucial for maintaining cellular equilibrium. The 70 kDa HSP (HSP70, encoded by the *HSPA1A* gene) provides cytoprotection against various stressors [22]. HSP70 acts as a principal molecular chaperone, facilitating numerous housekeeping and stress-response processes, which include the de novo folding and refolding of proteins, prevention of protein aggregation, targeted degradation of proteins, trafficking of transmembrane proteins, and the assembly or disassembly of protein complexes [23]. The chaperoning activity of HSP70 directly promotes cell survival by aiding the reassembly of compromised ribonucleoproteins [24]. In cancerous environments, metabolic stress (such as nutrient scarcity, hypoxia, and acidosis) leads to increased HSP70 expression, making it a critical target in cancer biology due to its role in mediating stress adaptation and therapy resistance [25]. Recent studies highlight the dual role of HSP70 in influencing both viral replication and anti-tumor immunity [26,27,28,29,30]. Nevertheless, the tactical manipulation of HSP70 signaling to improve oncolytic virotherapy remains largely unexplored.

The accumulation of reactive oxygen species (ROS) results in oxidative damage, leading to mitochondrial dysfunction and cellular injury [31]. At the same time, autophagy is triggered, wherein autophagosomes engulf damaged cellular components for subsequent breakdown in autolysosomes [32]. Importantly, ROS also function as signaling entities that launch the formation of autophagosomes and drive autophagic processes. Conversely, autophagy alleviates oxidative damage by eliminating protein aggregates and dysfunctional organelles, which in turn lowers ROS levels [33]. Herein, we demonstrate that a recombinant vaccinia virus armed with HSPA1A-targeting shRNA (oncoVV-shHSP70) dismantles ovarian cancer cells by enhancing autophagic flux and triggering an autophagy–ROS feedback loop, which then promotes inflammatory cytokine expression and viral replication. We also reveal that oncoVV-shHSP70 reshapes the tumor immune microenvironment (TIME) by expanding tumor-infiltrating hCD8^+^hGZMB^+^ T cells. These findings establish a paradigm-shifting approach to enhance oncolytic potency while overcoming immunosuppressive barriers in ovarian cancer.

## 2. Materials and Methods

### 2.1. Cell Culture

A2780 and SKOV3 human ovarian carcinoma cells, together with HEK293A embryonic kidney cells, were procured from China’s Meisen Cell Bank (Hangzhou, China). Prior to experiments, mycoplasma screening verified all cell lines were negative. Culture conditions consisted of DMEM with high glucose concentration (Gibco, Carlsbad, CA, USA), 10% FBS (Gibco, Carlsbad, CA, USA), and 1% penicillin/streptomycin (Sunribio, Hangzhou, China), with cells grown at 37 °C under 5% CO_2_.

### 2.2. Cell Viability Assay

The MTT colorimetric assay (Beyotime, Shanghai, China) was used to determine cell viability. Cells were cultured overnight in 96-well plates (NEST, Wuxi, China) to ensure adherence. Serial dilutions of oncoVV-shHSP70 or oncoVV (0.5–2 MOI) were applied, while PBS was used as a blank control. At 24, 48, and 72 h post-treatment, 20 μL of MTT solution (5 mg/mL) was added to each well and incubated for 4 h at 37 °C. After removing the supernatant, the formazan precipitate was dissolved in 150 μL of DMSO (Sigma, St. Louis, MO, USA). Absorbance at 490 nm was quantified using a microplate reader (Thermo Fisher, Waltham, MA, USA).

### 2.3. Construction and Preparation of Recombinant oncoVV-shHSP70

The oncoVV-shHSP70 was constructed as previously described [34]. An shRNA targeting HSPA1A was inserted into the plasmid pCB-023-II-Amp with *TK* gene deletion. The construct was packaged into viral particles by transfecting into PCB-293A cells (Thermo Fisher, Carlsbad, CA, USA). After infecting PCB-293A cells with wild type vaccinia virus (Western Reverse, WR) for approximately 2–4 h, pCB-023-II-shHSP70 was transfected into the cells. Vaccinia virus oncoVV-shHSP70 was harvested after 48 h.

The virus was then amplified in PCB-293A cells cultured in DMEM medium supplemented with 10% FBS. Viruses were harvested when cytopathic effect (CPE) exceeded 90%. After three freeze–thaw cycles and purification via cesium chloride gradient ultracentrifugation, the viral titer was determined by TCID_50_ assay [35]. The TCID_50_ titer was calculated using the Reed–Muench method, and then converted to PFU/mL based on the standard conversion (1 PFU ≈ 0.7 TCID_50_). The final infectious titer was determined using the formula PFU/mL = 7 × 10^(d+0.5)^, where “d” represents the weighted proportion of infected wells.

### 2.4. Virus Replication Assay

To monitor viral replication kinetics, SKOV3 cells were seeded in 24-well plates (NEST, Wuxi, China) at a density of 3 × 10^4^ cells per well and cultured for 8–12 h to allow adhesion. Experimental groups were treated with either 1 MOI (pre-titrated based on TCID_50_ titer, 1 MOI = 6.7 × 10^4^ TCID_50_/well) of viral inoculum, or viral inoculum combined with 150 µmol/L autophagy inhibitor 3-MA (Selleck, Houston, TX, USA), along with a PBS control group. Culture supernatants and cell lysates were collected at specified time points (24, 36, and 48 h). Viral quantification was performed using the end-point dilution assay: processed samples were centrifuged, then subjected to 10-fold serial logarithmic dilutions (10^−1^ to 10^−9^) in low-serum DMEM (2%). The diluted solutions were transferred to 96-well plates pre-seeded with HEK293A cells (3 × 10^3^ cells/well) and cultured for 7 days to observe cytopathic effects.

### 2.5. Xenograft Assays in Nude Mice

All animal procedures were approved by the Institutional Animal Care and Use Committee (IACUC) of Zhejiang Sci-Tech University (Protocol No. 202406071—27 May 2024) and were conducted in strict accordance with the Animal Research: Reporting of In Vivo Experiments (ARRIVE) guidelines to ensure scientific rigor and reproducibility. The xenograft model was developed in 4–6-week-old female Balb/c nude mice (Shanghai Slack Laboratory, Shanghai, China) in compliance with institutional animal care guidelines. Subcutaneous inoculation with 3 × 10^6^ A2780 cells was performed. Tumor-bearing mice were allocated to experimental groups when lesions reached 500–600 mm^3^. Therapeutic agents (100 μL of 1 × 10^7^ PFU/mL oncoVV or oncoVV-shHSP70) or saline control were delivered intratumorally. Tumor size was monitored every 4 days using the ellipsoid volume formula (0.5 × L × W^2^). Tumors at study endpoint were harvested and processed through 4% paraformaldehyde (Beyotime, Shanghai, China) fixation, graded alcohol dehydration, and paraffin embedding for microscopic evaluation.

### 2.6. Xenograft Assays in Humanized Mice

NOD.Cg-*Prkde^scid^Il2rg^tm1Sug^*/JicCrl (NOG) female mice aged 5–6 weeks (Vital River, Hangzhou, China) were used to establish the humanized mouse model. A total of 8 × 10^6^ human peripheral blood mononuclear cells (hPBMCs) were injected via the tail vein. Four days later, 1 × 10^7^ A2780 cells were subcutaneously inoculated into the humanized mice. When tumor volumes reached 100–200 mm^3^, mice were randomly divided into three groups (*n* = 4~6). Subsequently, 100 μL of oncoVV or oncoVV-shHSP70 (1 × 10^7^ PFU/mL) was intratumorally injected, with an equal volume of 0.9% NaCl as control. Tumor volumes were measured every three days. On day 21, mice were euthanized, and blood and tumor tissues were collected. Flow cytometry was performed to quantify hCD45, hCD3, and hCD8 cells. Remaining tumor tissues were fixed in 4% paraformaldehyde, dehydrated, and embedded in paraffin for histological evaluation.

### 2.7. Western Blot

Protein concentrations were determined using the BCA Protein Assay Kit (Vazyme, Nanjing, China). Following separation on 12% SDS-polyacrylamide gels, proteins were electrotransferred to PVDF membranes (Millipore, Burlington, MA, USA). Membranes were blocked for 1 h at RT with 5% non-fat milk (Solarbio, Beijing, China) before overnight incubation with primary antibodies at 4 °C. After thorough washing, secondary antibody incubation was performed for 1 h at room temperature. GAPDH was used as the internal control. Detection was achieved using the ECL detection kit (PerkinElmer, Waltham, MA, USA), with imaging conducted on the Clinx ChemiScope 6000 system (Clinx, Shanghai, China). Antibody information is available in Supplementary Table S1.

### 2.8. Flow Cytometric Analysis

Cells were treated with either oncoVV-shHSP70 or phosphate-buffered saline (PBS) before collection. Apoptosis assessment was performed with Annexin V-FITC and propidium iodide (PI) assay kits (BD Biosciences, Franklin Lakes, NJ, USA). For autophagy detection, we utilized the BBcellProbe^®^ M52 Autophagy Probe fluorescence staining kit (Bestbio, Nanjing, China). Reactive oxygen species (ROS) levels were quantified using the DCFH-DA kit (Solarbio, Beijing, China). All staining protocols followed the manufacturers’ instructions precisely. Cell analysis was conducted on a flow cytometer AccuriC6 (BD Biosciences, Franklin Lakes, USA), with data analysis performed in FlowJo™ V10.8 software (BD Life Sciences, Ashland, OR, USA).

Humanized mouse peripheral blood cells were separated using erythrocyte lysate (Solarbio, Beijing, China). Tumor specimens were washed with PBS, then dissociated using 0.25% trypsin solution (Solarbio, Beijing, China), stopped with serum-containing DMEM, and filtered to generate a single-cell suspension. Antibodies used were listed in Supplementary Table S2. Cellular analysis was performed using a flow cytometer Attune Nx (Thermo Fisher, Singapore), with FlowJo™ V10.8 software (BD Life Sciences, Ashland, OR, USA) for data processing.

### 2.9. Histological Examination

Tumor specimens were fixed, embedded in paraffin, and sliced into 4–6 μm sections. Following deparaffinization, tissue sections underwent graded alcohol rehydration before hematoxylin and eosin (H&E) staining. For IF analysis, slides were blocked with 3% BSA, incubated with primary antibodies overnight at 4 °C, washed with PBS, then treated with fluorescent secondary antibody (Supplementary Table S3) for 1 h, and counterstained with DAPI (Haoke Biotechnology, Hangzhou, China). Imaging was performed using an inverted fluorescence microscope (Nikon, Tokyo, Japan).

### 2.10. Quantitative Real-Time PCR

RNA extraction was conducted using the RNAprep Pure Cell/Bacteria Kit (Tiangen Biotech, Beijing, China) following standard procedures. Subsequently, 1 μg of RNA was reverse-transcribed into cDNA using the PrimeScript™ RT Reagent Kit (Takara, Shiga, Japan). Quantitative real-time PCR (qPCR) was executed with AceQ^®^ qPCR SYBR Green Master Mix (Vazyme, Nanjing, China) on the LineGene 9600 system (Bioer, Hangzhou, China). Glyceraldehyde 3-phosphate dehydrogenase (GAPDH) was employed as the reference gene. The 2-ΔΔCt method was applied to compute relative expression, with fold changes derived from exponential transformation of Ct values. Primer sequences are detailed in Supplementary Table S4.

### 2.11. Statistical Analysis

Data were analyzed using GraphPad Prism (version 9) software. All data are presented as the mean ± standard deviation (SD). For comparisons between two groups, the Mann–Whitney U test was utilized. For comparisons among three or more groups, one-way analysis of variance (ANOVA) was performed, followed by Tukey’s post-hoc test. A *p*-value of less than or equal to 0.05 (*p* ≤ 0.05) was considered statistically significant.

## 3. Results

### 3.1. The Anti-Tumor Effect of oncoVV-shHSP70 Both In Vivo and In Vitro

**Figure 1:**
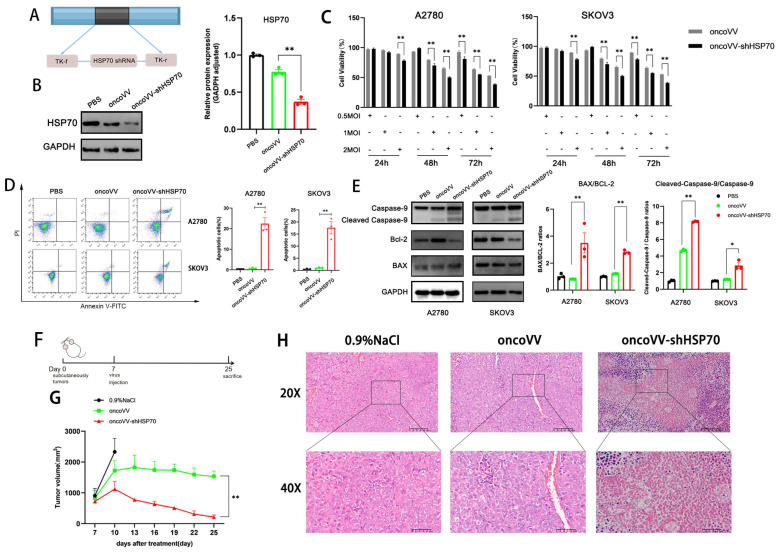
OncoVV−shHSP70 exhibits potent anti−tumor effects in ovarian cancer (OV) cells. (**A**) Schematic structure of oncoVV−shHSP70. (**B**) Validation and quantification of HSP70 knockdown in constructed virus (oncoVV−shHSP70) by Western blot; (**C**) MTT assay was performed to measure the cell viability of A2780 and SKOV3 cells after infection with oncoVV or oncoVV−shHSP70 at the indicated multiplicities of infection (MOI: 0.5, 1, 2); (**D**) Apoptosis rates of A2780 and SKOV3 cells were assessed by flow cytometry; (**E**) The expression of apoptosis−related proteins was detected using Western blot. (**F**) Timeline of the mouse experiment; (**G**) The volume of xenograft tumors; (**H**) HE staining of tumor tissues. Data are presented as mean ± SD. Significance among three groups was determined by one-way ANOVA followed by Tukey’s post-hoc test. (* *p* ≤ 0.05, ** *p* ≤ 0.01).

The recombinant oncolytic vaccinia virus oncoVV-shHSP70 was generated from the WR strain by deleting the *TK* region and inserting a short hairpin RNA (shRNA) targeting *HSPA1A* (Figure 1A). Successful knockdown of HSP70 was confirmed by Western blotting (Figure 1B). To evaluate cytotoxicity in ovarian cancer cells, A2780 and SKOV3 human ovarian carcinoma cells were treated with oncoVV control or oncoVV-shHSP70 at MOI of 0.5, 1, and 2. MTT assays at 24, 48, and 72 h post-infection revealed that oncoVV-shHSP70 caused significantly enhanced growth inhibition in a dose- and time-dependent manner compared to the oncoVV control (Figure 1C).

Building upon our previous studies demonstrating oncoVV-induced apoptosis across malignancies, this study investigated apoptosis in ovarian cancer. Annexin V-FITC/PI staining quantified by flow cytometry revealed significant increases in apoptotic cells in oncoVV-shHSP70-treated groups compared to oncoVV control and PBS control (11–30-fold increase, Figure 1D). Mechanistically, immunoblotting showed enhanced activation of the mitochondrial apoptotic pathway, evidenced by elevated cleaved caspase-9 and an increased Bax/Bcl-2 ratio (Figure 1E). These findings indicate that HSP70 silencing potentiates oncoVV-induced mitochondrial apoptosis in ovarian cancer cells.

The anti-tumor efficacy was further assessed in A2780 xenograft-bearing nude mice (Figure 1F). Consistent with in vitro results, intratumoral injection of oncoVV-shHSP70 exhibited significantly stronger tumor growth suppression compared to oncoVV or 0.9% NaCl control (Figure 1G). H&E staining showed extensive tumor necrosis in oncoVV-shHSP70-treated tumors (Figure 1H).

### 3.2. OncoVV-shHSP70 Orchestrates an ROS–Autophagy Feedback Loop

Cell death is an essential physiological process, which encompasses the classical forms of apoptosis, autophagy, and necrosis, which are now understood not as isolated pathways but as interconnected programs whose interplay determines the final cellular outcome [36,37]. To investigate whether autophagy facilitates oncoVV-shHSP70-induced cell death, we analyzed autophagosome dynamics by utilizing the M52 probe (Bestbio, China) in conjunction with flow cytometric assessments. Remarkably, treatment with oncoVV-shHSP70 resulted in a 4–5.7-fold rise in autophagic activity compared to the parental virus control (Supplementary Figure S1A). Immunoblot analysis indicated the activation of autophagic flux, evidenced by an increased LC3-II/LC3-I ratio and reduced p62 levels at 36 h post-infection (Supplementary Figure S1B). Viral titration using the TCID50 assay indicated a 5.5-fold enhancement in viral titers with oncoVV-shHSP70 compared to oncoVV (1.2 × 10^7^ vs. 2.2 × 10^6^ PFU/mL). Importantly, this increase was significantly inhibited by co-treatment with the autophagy inhibitor 3-MA (Supplementary Figure S1C), demonstrating that the pro-replicative effect of oncoVV-shHSP70 depends on autophagic flux. This suggests that the virus promotes viral replication by inducing autophagy.

**Figure 2:**
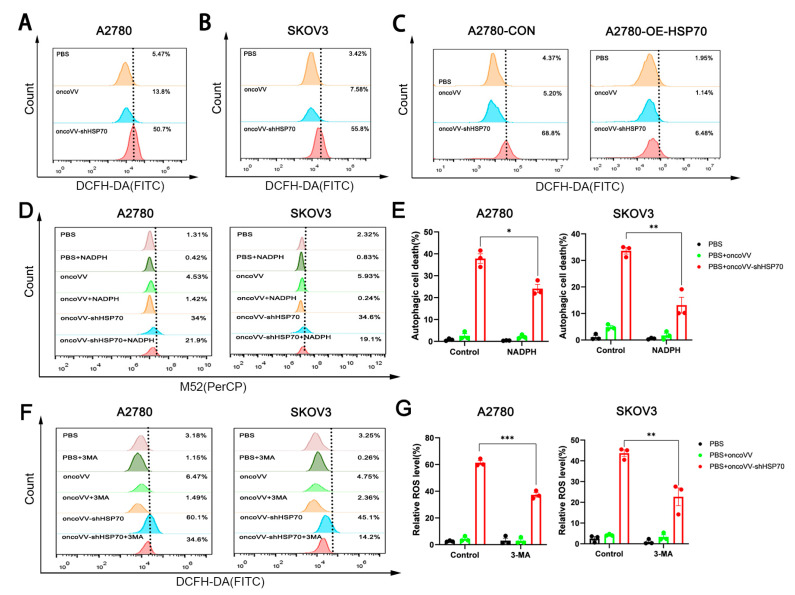
OncoVV-shHSP70 induces ROS production and orchestrates ROS−autophagy crosstalk. (**A**) ROS levels in A2780 cells were detected through DCFH-DA fluorescent probe and measured by flow cytometry; (**B**) ROS levels in SKOV3 cells detected; (**C**) Overexpressed HSP70 significantly inhibits ROS levels in A2780 cells; (**D**) NADPH significantly suppresses autophagy in OV cells; (**E**) Quantification of autophagic cell death rates after NADPH treatment; (**F**) 3−MA significantly inhibits ROS levels in OV cells; (**G**) Quantification of relative ROS levels after 3−MA treatment. Data are presented as mean ± SD. Significance among three groups was determined by one-way ANOVA followed by Tukey’s post-hoc test. (* *p* ≤ 0.05, ** *p* ≤ 0.01, *** *p* ≤ 0.001).

ROS, extensively studied in cancer due to their ability to damage proteins, nucleic acids, lipids, membranes, and organelles (inducing cell death), show therapeutic promise in both in vitro and in vivo settings [38]. To explore the potential of ROS modulation by oncoVV-shHSP70 in ovarian cancer cells, ROS levels were measured. Flow cytometry using DCFH-DA revealed 3.7-fold (A2780) and 7.4-fold (SKOV3) increases in intracellular ROS levels in oncoVV-shHSP70-treated cells versus oncoVV controls (Figure 2A,B). Critically, HSP70-overexpressing A2780 cells (A2780-OE-HSP70) exhibited complete suppression of ROS production by oncoVV-shHSP70 (Figure 2C), confirming oncoVV− shHSP70’s central role in triggering ROS accumulation. Strikingly, the ROS scavenger NADPH significantly attenuated oncoVV-shHSP70-induced autophagic activity by 35.6–44.8% (Figure 2D,E). Conversely, the autophagy inhibitor 3-MA suppressed the viral-induced ROS surge by 42.4–68.5% (Figure 2F,G). This bidirectional modulation establishes a crosstalk between ROS and autophagy, and positions ROS not merely as cytotoxic byproducts, but as essential signaling molecules coordinating viral replication fidelity through redox-sensitive autophagic adaptation.

### 3.3. OncoVV-shHSP70 Activates Antitumor Inflammation

**Figure 3:**
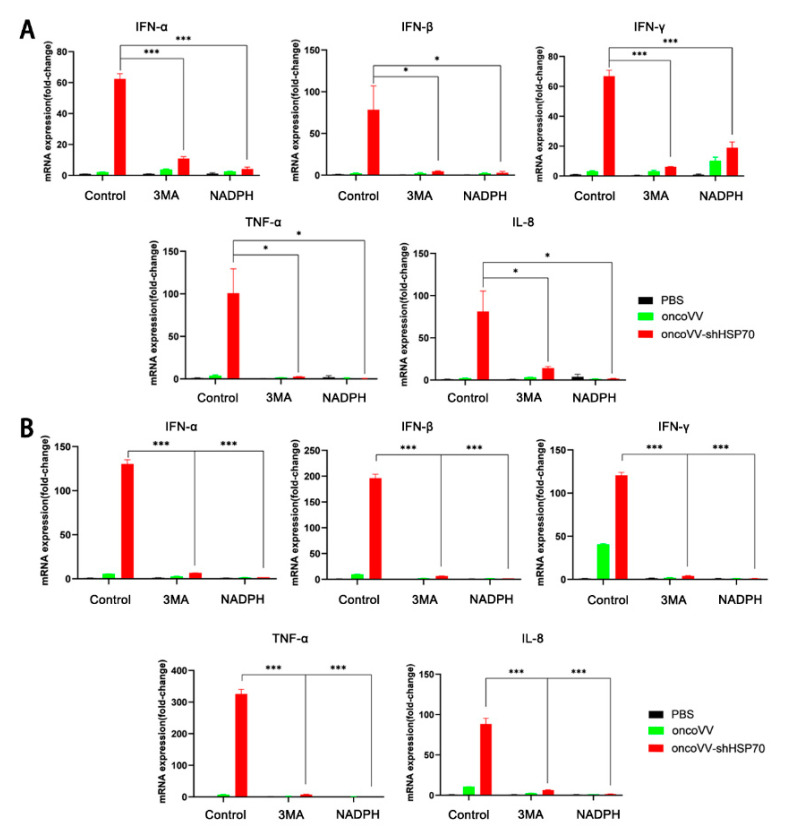
OncoVV−shHSP70 promotes the transcription of inflammatory cytokines, while NADPH and 3−MA inhibit the promotion. (**A**) The mRNA levels of IFN−α, IFN−β, IFN−γ, TNF−α, and IL−8 in A2780 cells were detected by RT−qPCR assay; (**B**) The mRNA levels of IFN−α, IFN−β, IFN−γ, TNF−α, and IL−8 in SKOV3 cells detected by RT−qPCR. Data are presented as mean ± SD. Significance among multiple groups was determined by one−way ANOVA followed by Tukey’s post−hoc test. (* *p* ≤ 0.05, *** *p* ≤ 0.001).

Growing evidence indicates that oncolytic viruses elicit anti-tumor immune responses in the host [39,40], consistent with emerging paradigms of viral-mediated immunogenic cell death [41]. To determine whether oncoVV-shHSP70 drives this immunogenic response, we interrogated inflammatory activation in treated ovarian cancer cells. RT−qPCR revealed that transcription levels of pro-inflammatory cytokines including IL-8, TNF−α, IFN−α, IFN−β and IFN−γ were significantly elevated by 50–300-fold in oncoVV-shHSP70−treated ovarian cancer cell lines compared to controls (Figure 3A, B). Notably, this induction was substantially suppressed by either 3−MA or NADPH, indicating that the ROS−autophagy loop modulates this inflammatory cascade. Critically, such modulation contributes to the anti-tumor efficacy of oncoVV-shHSP70. Collectively, these data demonstrate that the autophagy−ROS feedback loop enhances the anti−tumor efficacy of oncoVV−shHSP70 by amplifying both viral replication and inflammatory cytokine expression.

Interestingly, under our experimental conditions, the parental oncoVV control did not elicit a significant interferon response. This differential induction profile may be attributed to the specific viral tropism and replication kinetics of the vaccinia virus strain used, which can result in cell−type−dependent modulation of innate immune signaling. Nevertheless, the data clearly establish that HSP70 silencing is sufficient to unlock a robust antiviral and inflammatory gene program.

### 3.4. OncoVV-shHSP70 Remodels TIME via Cytotoxic CD8^+^ T−Cell Recruitment in Humanized Mice

**Figure 4:**
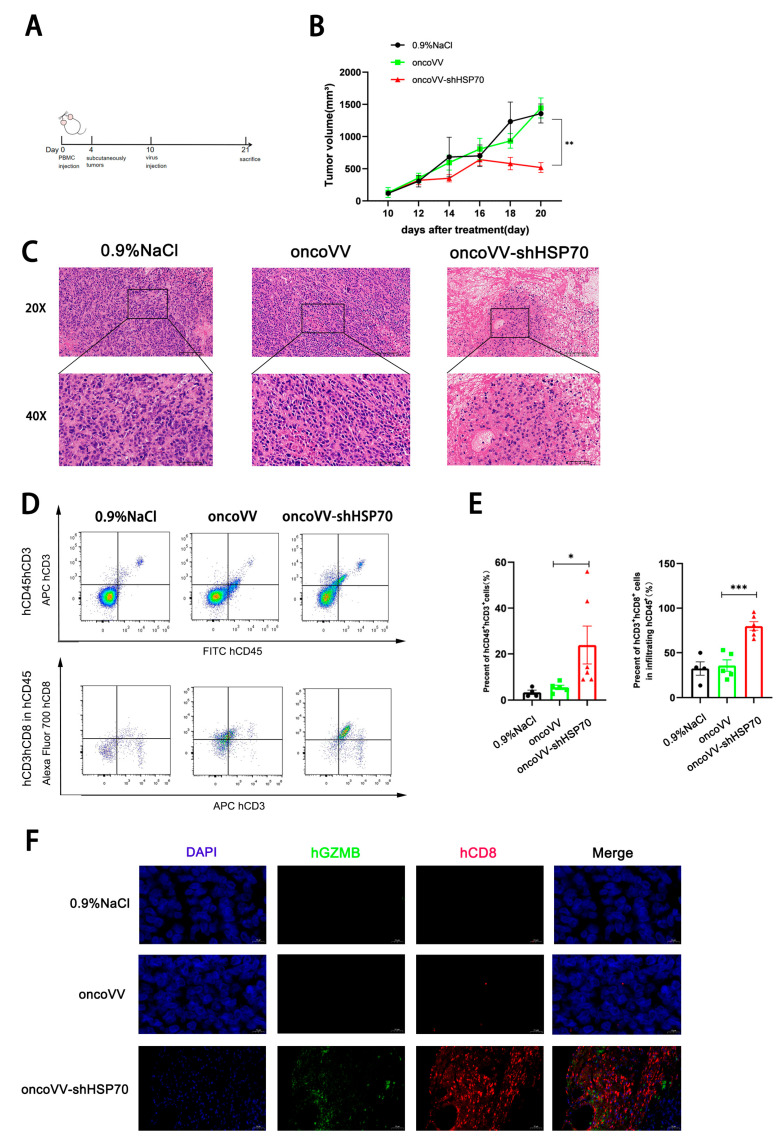
OncoVV−shHSP70 activating tumor infiltration T cells in humanized mouse model. (**A**) Timeline of the humanized mouse experiment; (**B**) The volume of xenograft tumors was measured every 2 days; (**C**) H&E staining of tumor tissues; (**D**,**E**) The percentages of hCD3^+^hCD8^+^hCD45^+^ cells in xenograft tumors were detected and quantified by flow cytometry; (**F**) Immunofluorescence observation of hCD8^+^hGZMB^+^ cells in xenograft tumors. Blue, DAPI staining, Green, GZMB staining, Red, CD8 staining. Data are presented as mean ± SD. Significance among three groups was determined by one-way ANOVA followed by Tukey’s post-hoc test. (* *p* ≤ 0.05, ** *p* ≤ 0.01, *** *p* ≤ 0.001).

Having established the oncoVV−shHSP70-induced autophagic flux and ROS amplification as key effectors of direct tumor lysis, we next interrogated its capacity to remodel the TIME for coordinated immunotherapy. To dissect the oncoVV−shHSP70−induced TIME remodeling, we established tumor−bearing humanized mice (Figure 4A). Tumor volume monitoring demonstrated that oncoVV−shHSP70 significantly inhibited tumor growth in humanized mice (Figure 4B). H&E staining indicated extensive structural damage in treated tumors by oncoVV−shHSP70 (Figure 4C). To investigate the effect of oncoVV−shHSP70 on the immune cell composition of the TIME, we used flow cytometry to analyze immune cell profiles in single−cell suspensions generated from xenograft tumors. The result revealed that oncoVV−shHSP70 markedly increased the proportion of tumor-infiltrating hCD3^+^hCD8^+^hCD45^+^ cells (Figure 4D,E). Immunofluorescence observation confirmed a pronounced increase in hCD8^+^hGZMB^+^ cells within tumors treated with oncoVV−shHSP70 (Figure 4F). Collectively, oncoVV-shHSP70 reshapes the tumor immune microenvironment by enhancing hCD8^+^ hGZMB^+^ T cell infiltration.

### 3.5. OncoVV-shHSP70 Modulates Systemic T Cell Distribution in Humanized Mice

**Figure 5:**
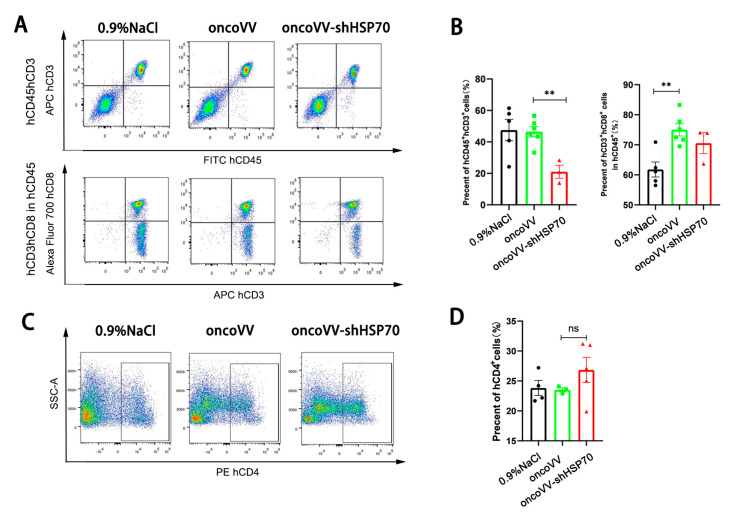
OncoVV-shHSP70 activates peripheral blood immune cells in a humanized mouse model. (**A**,**B**) The percentages of hCD3^+^hCD8^+^hCD45^+^ cells in mouse peripheral blood were detected by flow cytometry; (**C**,**D**) The percentages of hCD4^+^ cells in mouse peripheral blood were detected by flow cytometry. Data are presented as mean ± SD. Significance among three groups was determined by one-way ANOVA followed by Tukey’s post-hoc test. (ns *p* > 0.05, ** *p* ≤ 0.01).

Peripheral blood analysis revealed that oncoVV−shHSP70, but not the control virus, significantly reduced circulating hCD3^+^hCD45^+^ T cell proportions (Figure 5A,B). In contrast, the proportion of hCD4^+^ T cells remained unaltered (Figure 5C,D). This pattern is consistent with the active trafficking of cytotoxic T cells from the periphery to the tumor site. This specific reduction in cytotoxic T cells, coupled with their concurrent accumulation in tumors, indicates their active trafficking from the periphery to the tumor site. This trade−off highlights the pivotal role of CD8^+^ T cell recruitment in the anti−tumor efficacy of oncoVV−shHSP70.

## 4. Discussion

The efficacy of OVs was historically attributed primarily to their direct oncolytic capacity, where intratumoral replication and dissemination of progeny virions lead to cancer cell death [42]. However, it is now evident that OVs act through more complex mechanisms. By inducing immunogenic cell death (ICD), they release damage− and pathogen-associated molecular patterns (DAMPs/PAMPs). This process promotes a pro−inflammatory cytokine response and activates NK cells and CD8^+^ T cells, initiating potent antigen-specific anti−tumor immunity [43,44,45,46].

**Figure 6:**
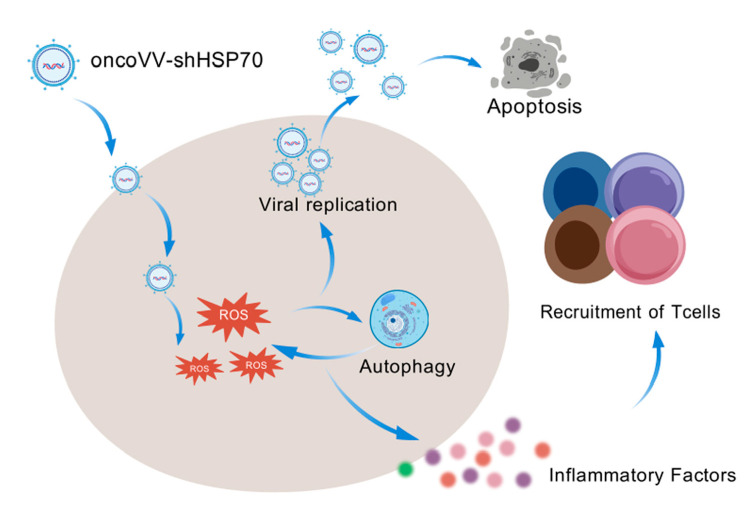
Schematic of the mechanism-of-action of oncoVV-shHSP70 in ovarian cancer cells.

Our previous research demonstrated that recombinant oncolytic vaccinia viruses effectively induce tumor cell death by promoting both viral replication and an inflammatory response [15,17,47]. This investigation reveals that silencing HSP70 in conjunction with oncolytic vaccinia virus (oncoVV−shHSP70) results in enhanced efficacy against ovarian cancer through the synergistic activation of the intrinsic apoptotic pathway, increasing autophagic flux and ROS generation, and establishing a ROS−autophagy feedback loop that regulates viral replication and pro−inflammatory cytokine expression (Figure 6). This positions HSP70 silencing as a strategy to disrupt proteostasis and enhance oncolytic virotherapy.

It is well−recognized that HSP70 has been a target of longstanding interest in cancer therapeutics. HSP70 is overexpressed in various cancers, supporting tumor progression through diverse pro-oncogenic activities [48,49,50]. In light of this clinical relevance, both small-molecule inhibitors specifically targeting HSP70 [51,52] and immunotherapy approaches based on HSP70 have progressed to clinical trial stages [11,53,54]. In contrast to small molecule inhibitors that directly antagonize HSP70’s chaperone activity, our strategy of silencing HSP70 expression in tumor cells using an oncolytic virus triggers a unique cascade of events−a self−amplifying autophagy−ROS feedback loop−that directly enhances viral replication and promotes immunogenic cell death. This regulatory mechanism positions the crosstalk between ROS and autophagy as a modifiable enhancer of viral oncolysis, thereby broadening the anti-tumor actions of recombinant vaccinia viruses beyond traditional immunomodulation. Furthermore, our platform simultaneously remodels the tumor immune microenvironment, a multi-modal anti-tumor effect not achievable with small molecule inhibitors alone.

The TIME significantly influences immunotherapy response. Tumors with limited T-cell infiltration (‘cold’ tumors) are typically resistant [55,56]. Our data in humanized mice models demonstrated that oncoVV-shHSP70 treatment significantly increased the infiltration of cytotoxic hCD8^+^hGZMB^+^ T cells into tumors, suggesting its potential to favorably alter the TIME. This effect was accompanied by a reduction in corresponding T cell subsets in the peripheral blood. Therefore, the concomitant reduction in peripheral T cells likely reflects their active recruitment to the tumor site, supporting the notion of productive trafficking rather than simple depletion. This repositioning of T cells highlights a key immunotherapeutic mechanism of oncoVV−shHSP70.

Collectively, this study positions HSP70 as a key regulator of the oncolysis−immunity continuum. The oncoVV−shHSP70 strategy, leveraging the autophagy–ROS feedback loop and immune activation, opens new frontiers for viro−immunotherapy in solid tumors like ovarian cancer. Future challenges include optimizing delivery to metastatic sites and assessing the safety profile of HSP70 modulation.

## Data Availability

The original contributions presented in this study are included in the article/Supplementary Material. Further inquiries can be directed to the corresponding authors.

## Supplementary Materials

The following supporting information can be downloaded at: https://www.mdpi.com/article/10.3390/v17111423/s1, Figure S1: OncoVV-shHSP70 induces autophagic flux and enhances viral replication; Table S1: Antibodies for Western blot; Table S2: Antibodies for Flow Cytometry; Table S3: Antibodies for Immunohistochemical staining; Table S4: Primers for qPCR.
