# Supplementary material for: Oncolytic Vaccinia Virus Expressing HSP70 shRNA Exerts Anti-Tumor Effects in Human Ovarian Cancer via Triggering the Autophagy–ROS Feedback Loop and Immune Activation
Source: Viruses. 2025 Oct 27;17(11):1423. doi: 10.3390/v17111423 (PMC12656899; doi:10.3390/v17111423)
Supplement: Supplementary file 1 [file viruses-17-01423-s001.zip › viruses-3887882-supplementary.pdf]

Supplementary for

Oncolytic vaccinia virus harboring HSP70 shRNA exerts anti-tumor effects by triggering apoptotic/autophagic flux and activating CD8⁺ T cells in human ovarian cancer

Zheqi Cai, Zhiyun Hong, Guohui Zhang, Tinwei Zhu, Yanrong Zhou, Ting Ye, Gongchu Li, Kan Chen

Supplementary Figure S1.

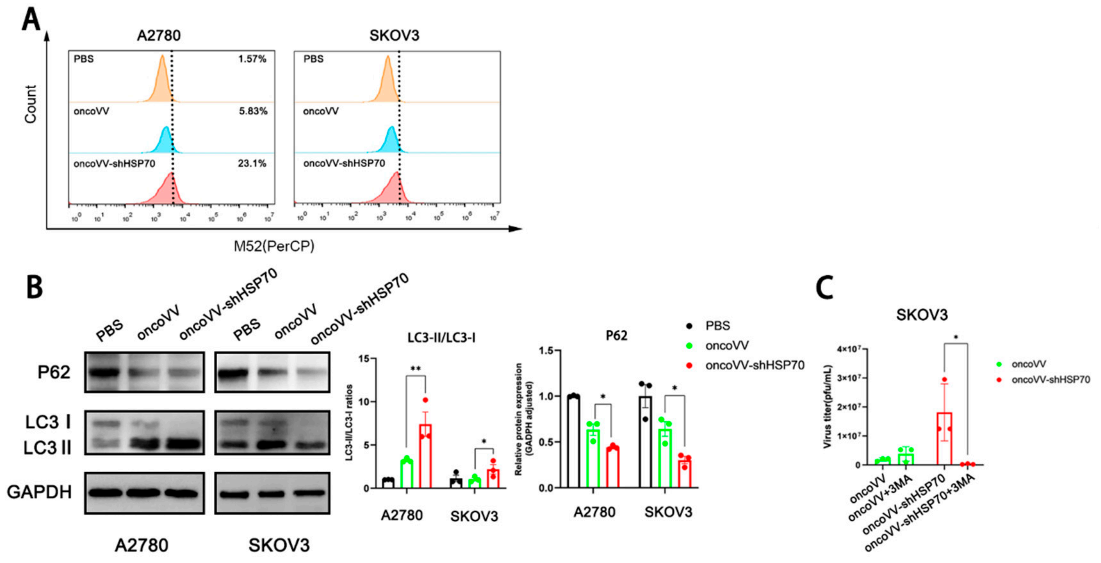

Figure S1. OncoVV-shHSP70 induces autophagic flux and enhances viral replication. (A) Flow cytometric analysis of autophagic activity in A2780 and SKOV3 cells stained with the M52 autophagosome probe after treatment. (B) Western blot analysis of LC3 and p62 protein levels, with quantification demonstrating an increased LC3-II/LC3-I ratio and decreased p62, indicating enhanced autophagic flux. (C) Viral titers were determined by $TCID_{50}$ assay, showing that the autophagy inhibitor 3-MA significantly suppressed oncoVV-shHSP70 replication in A2780 and SKOV3 cells. Data are presented as mean ± SD. Significance was determined by one-way ANOVA followed by Tukey's post-hoc test. (*p ≤ 0.05, **p ≤0.01).

Supplementary Table S1

Antibodies for Western blot

Target	Host Species	Dilution	Distributed By	Product Nr
Caspase-9	Rabbit	1：4000	Cell Signaling Technology, Inc., Danvers, MA, USA	9502
Bax	Rabbit	1：4000	Cell Signaling Technology, Inc., Danvers, MA, USA	2772

GAPDH	Rabbit	1：4000	Cell Signaling Technology, Inc., Danvers, MA, USA	2118
BCL-2	Mouse	1：4000	Cell Signaling Technology, Inc., Danvers, MA, USA	15071s
HSP70	Mouse	1：4000	Cell Signaling Technology, Inc., Danvers, MA, USA	46477s
P62	Rabbit	1：4000	Cell Signaling Technology, Inc., Danvers, MA, USA	39749S
LC3A/B	Rabbit	1：4000	Cell Signaling Technology, Inc., Danvers, MA, USA	12741T

Supplementary Table S2

Antibodies for Flow Cytometry

Usage	Host Species	Dilution	Distributed By	Product Nr
APC anti-human CD3 Antibody	Mouse	1：20	BioLegend, Inc., San Diego, CA, USA	317318
FITC anti-human CD45 Antibody	Mouse	1：20	BioLegend, Inc., San Diego, CA, USA	304006
Alexa Fluor® 700 anti-human CD8 Antibody	Mouse	1：20	BioLegend, Inc., San Diego, CA, USA	344724
PE anti-human CD4 Recombinant Antibody	Mouse	1：20	BioLegend, Inc., San Diego, CA, USA	302404

Supplementary Table S3

Antibodies for Immunohistochemical staining

Usage	Host species	Dilution	Distributed By	Product Nr
CD8a Polyclonal antibody	Rabbit	1:1000	Proteintech Group, Inc., Rosemont, IL, USA	29896-1-ap
Anti-Granzyme B Antibody	Rabbit	1:1000	Abcam plc, Cambridge, UK	ab255598
Ready-to-use Poly-HRP Goat Anti-Rabbit Secondary Antibody	Rabbit	1：1	Haokebio, Inc., Shanghai, China	HKI0026

Supplementary Table S4

Primers for qPCR

Primers	Sequence	Usage
IFN-γ	IFN-γ-F: TCAGCTCTGCATCGTTTTGG<br>IFN-γ-R: GTTCCATTATCCGCTACATCTGAA	qPCR
IFN-β	IFN-β-F: CAGCAATTTTCAGTGTCAGAAGC	qPCR

	IFN-β-R: TCATCCTGTCCTTGAGGCAGT	
IFN-α	IFN-α-F: GTGAGGAAATACTTCCAAAGAATCAC<br>IFN-α-R: TCTCATGATTTCTGCTCTGACAA	qPCR
TNF-α	TNF-α-F: CTAAGAGGGAGAGAAGCAACTAC<br>TNF-α-R: TCAGTATGTGAGAGGAAGAGAAC	qPCR
IL-8	IL-8-F: GAGAGTGATTGAGAGTGGACCAC<br>IL-8-R: CACAACCCTCTGCACCCAGTTT	qPCR
GAPDH	GAPDH-F: GACAGTCAGCCGCATCTTCT<br>GAPDH-R: GCGCCCAATACGACCAAATC	qPCR
